# Survival of human embryonic stem cells implanted in the guinea pig auditory epithelium

**DOI:** 10.1038/srep46058

**Published:** 2017-04-07

**Authors:** Min Young Lee, Sandra Hackelberg, Kari L. Green, Kelly G. Lunghamer, Takaomi Kurioka, Benjamin R. Loomis, Donald L. Swiderski, R. Keith Duncan, Yehoash Raphael

**Affiliations:** 1 Kresge Hearing Research Institute, Otolaryngology - Head and Neck Surgery, The University of Michigan Medical School, MSRB-3, Rm. 9301 1150 W. Medical Center Dr. Ann Arbor, MI 48109-5648, USA; 2 Department of Otorhinolaryngology and Head & Neck Surgery, Dankook University Hospital, 119, Dandae-ro, Dongnam-gu, Cheonan-si, Chungnam, 31116, Korea.

## Abstract

Hair cells in the mature cochlea cannot spontaneously regenerate. One potential approach for restoring hair cells is stem cell therapy. However, when cells are transplanted into scala media (SM) of the cochlea, they promptly die due to the high potassium concentration. We previously described a method for conditioning the SM to make it more hospitable to implanted cells and showed that HeLa cells could survive for up to a week using this method. Here, we evaluated the survival of human embryonic stem cells (hESC) constitutively expressing GFP (H9 Cre-LoxP) in deaf guinea pig cochleae that were pre-conditioned to reduce potassium levels. GFP-positive cells could be detected in the cochlea for at least 7 days after the injection. The cells appeared spherical or irregularly shaped, and some were aggregated. Flushing SM with sodium caprate prior to transplantation resulted in a lower proportion of stem cells expressing the pluripotency marker Oct3/4 and increased cell survival. The data demonstrate that conditioning procedures aimed at transiently reducing the concentration of potassium in the SM facilitate survival of hESCs for at least one week. During this time window, additional procedures can be applied to initiate the differentiation of the implanted hESCs into new hair cells.

The hair cells in the cochlea transduce sound to initiate signaling leading to hearing. These hair cells in the mature mammalian cochlea are not spontaneously replaced when lost, resulting in permanent hearing loss^1^,^2^,^3^. If the loss of hair cells is severe or complete, the deafness is profound and the only feasible clinical management relies on a cochlear implant, which does not replicate the sound quality of the normal cochlea. Novel biological approaches for restoration of hair cells may provide better hearing than the cochlear implant. Possible methods include the transdifferentiation of supporting cells into hair cells and transplantation of stem cells into the cochlea followed by stepwise differentiation into new hair cells. Transplantation is especially important when endogenous supporting cells are flat and non-responsive to transdifferentiation protocols, and in cases of genetic disease where exogenous wild-type cells not bearing the mutation may provide the cure.

Protocols for generating inner ear progenitors^4^,^5^,^6^,^7^ or inner ear organoids^8^,^9^,^10^ from stem cells have been reported. The next step towards the usage of stem cells as a replacement for lost hair cells should address the transplantation of the cells into the cochlea, their survival and integration into the tissue, and their guided differentiation into hair cells *in vivo*. This task is complicated for several reasons. Following delivery of cells into the scala tympani, a fluid-filled lumen adjacent to the cochlear duct epithelium, transplanted cells can survive but remain in the perilymph in scala tympani and do not reach the auditory epithelium where they need to reside to transduce sound efficiently. Consequently, it is necessary to inject the cells directly into the scala media, the fluid-filled lumen within the cochlear duct epithelium. However, the high concentration of potassium of the endolymph^11^ in scala media quickly kills the transplanted cells^12^,^13^. The two-fold task is therefore to generate conditions that will allow transplanted cells to survive in the endolymph, and then to integrate these cells into the auditory epithelium, an epithelial sheet with especially robust apical junctions^14^,^15^,^16^,^17^. To promote cell survival and integration, endolymph can be transiently “conditioned” to decrease the potassium concentration and open cell-cell junctions in the auditory epithelium. Using this conditioning method, we showed that HeLa cells injected into scala media could survive for at least one week^13^.

Human embryonic stem cells (hESC) can be maintained in culture and are amendable to molecular manipulation^18^. They are pluripotent stem cells that are able to differentiate into all cell types in the human body^19^,^20^,^21^ including hair cell-like cells^5^,^6^,^9^,^22^,^23^,^24^ *in vitro*. In this study, we tested whether the conditioning is sufficient to make the endolymph hospitable to cells less robust than the HeLa cells used in our previous experiments. Specifically, we tested the survival of hESCs in the conditioned deaf guinea pig cochleae, and the influence of the cochlear environment on the subsequent differentiation of the hESCs.

## Results

### Pluripotency and GFP expression of stem cells *in vitro*

**Figure 1:**
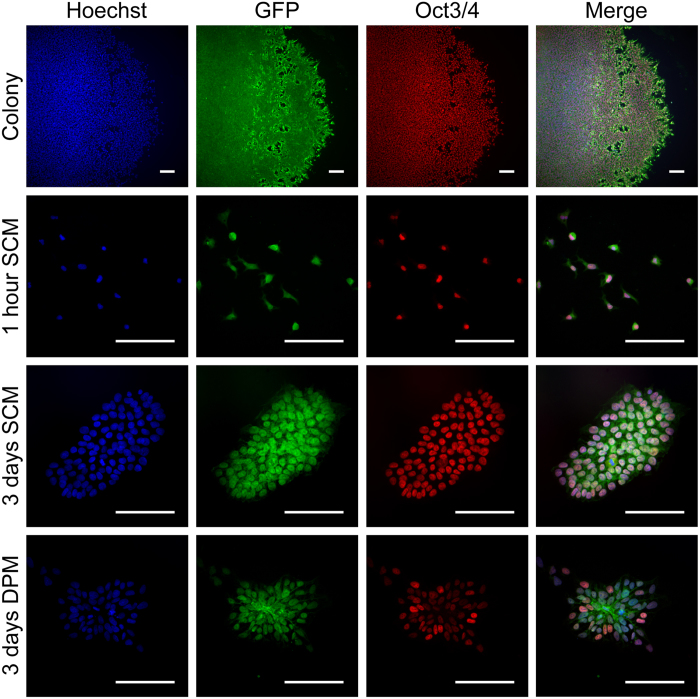
hESC pluripotency and GFP expression. Representative images of hESC expression of GFP and the pluripotency marker Oct3/4 in hESCs maintained in colonies, 1 h after plating single cells in the stem cell maintenance medium (SCM) TeSR-E8 and 3 days after single cell generation and culture in SCM or serum free DPM. Scale bars indicate 100 μm in all images.

To facilitate detection of transplanted stem cells, we used a modified human embryonic stem cell line (H9 Cre-LoxP) that constitutively expresses GFP. Cultures of these hESCs in stem cell maintenance medium (SCM) produced characteristic growth in rounded colonies with homogeneous expression of GFP and the pluripotency marker Oct3/4 (Fig. 1). Because dissociation into single cell suspensions—as required for later transplantation—can influence differentiation, we examined the persistence of GFP and Oct3/4 expression 1 hour, 1 day, and 3 days after dissociation and transfer to SCM or differentiation permissive medium (DPM). Robust GFP expression was observed at all time points in both SCM and DPM conditions; Oct3/4 was maintained in SCM cultures but was lost in many cells cultured in DPM for 3 days (Fig. 1). Overall, at least 95% of these cells were positive for Oct3/4 under SCM culture conditions, whereas DPM cultures at 3 days exhibited irregularly shaped colonies with Oct3/4-negative single cells present along the colony borders, indicative of differentiation. Both differentiating and pluripotent cells were GFP-positive, facilitating the identification of these cells in the xenografts described below.

### ABR threshold and histologic changes after deafening or conditioning

**Figure 2:**
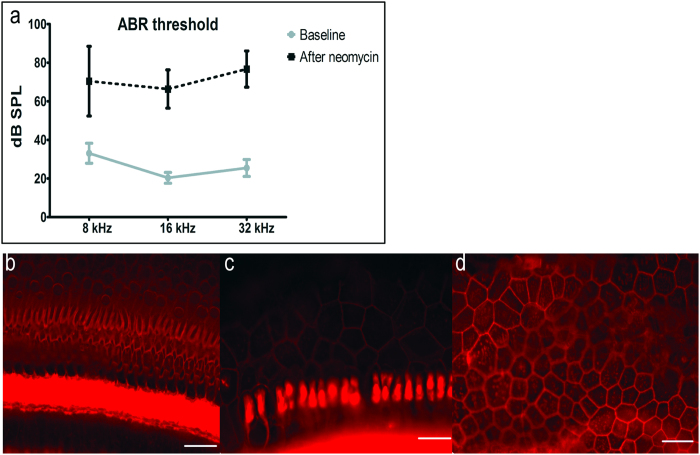
ABR thresholds and histologic changes after unilateral neomycin deafening. (**a**) There was statistically significant elevation of thresholds overall (MANOVA, p < 0.01) and at each tested frequency (p < 0.05). Epi-fluorescence of auditory epithelium stained by phalloidin (actin, red) showed diverse tissue morphologies: scar formation (**b**), transition from scar formation to flat epithelium (**c**) or flat epithelium (**d**), but always complete hair cell loss. Scale bars are 25 μm.

Clinically, candidate ears for stem cell therapy will have profound deafness due to a complete loss of hair cells. Therefore, we used deafened guinea pigs with widespread hair cell loss and profound deafness. We confirmed the outcome of the deafening insult using ABRs, measured one week after the unilateral deafening surgery. Statistically elevated ABR thresholds were obtained after deafening with neomycin (MANOVA; F = 174.79, df = 3, *p* < 0.001). Deafened animals had elevated thresholds at all frequencies (Fig. 2a). Residual hearing function was observed and probably is due to transmission of the sound stimulus to the undamaged (right) ear^25^. In the left ears, hair cells were invariably eliminated by the procedure. Supporting cells responded with differing degrees of pathological changes. In some areas, supporting cells remained in their normal height and exhibited the typical phalangeal scar formation (Fig. 2b). In other areas, we observed a transitional stage between scar formation and a flat epithelium (Fig. 2c), or flat epithelium (Fig. 2d).

Two undeafened animals were used to measure ABRs and organ of Corti histology after the conditioning protocol. In these animals, ABR assays could detect hearing 4 days after the conditioning procedure. Because hearing cannot be elicited by sound when endocochlear potential (EP) is completely absent, our result indirectly indicates that the stria vascularis can recover from the furosemide and generate an EP. Compared to normative data obtained from hundreds of guinea pigs, the threshold shifts exhibited in these non-deafened and conditioned ears were 43 and 51 dB at 4 kHz and 23 and 58 dB at 20 kHz. These two cochleae were assessed morphologically and were found to have significant outer hair cell loss in the region of the injection but the area closer to the apical end of the cochlea appeared nearly normal (Suppl. Fig. 1).

### The characteristics of transplanted hESCs within the scala media

**Figure 3:**
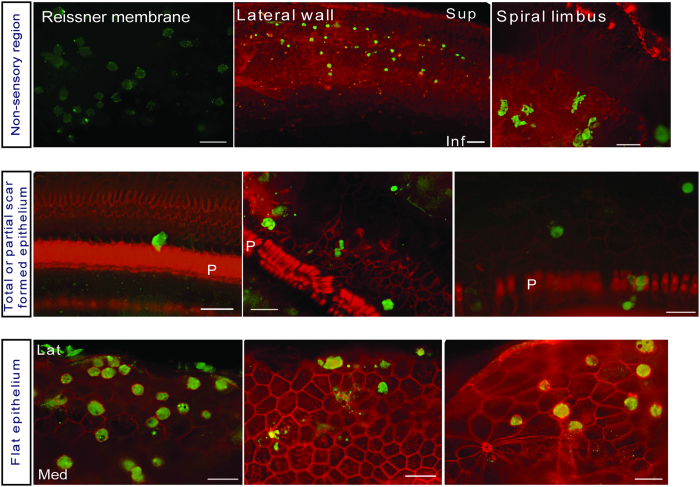
Locations of transplanted hESCs in the scala media 1 day after injection. Whole mounts showing presence of hESCs in non-sensory regions (top row), and in sensory regions with scar formation (middle row) or flat epithelium (bottom row). Locations of hESCs are indicated by self-expression of GFP (green); tissues were counterstained with phalloidin to indicate actin in cell junctions (red). In the sensory epithelium, a larger number of hESCs was observed in the flat epithelium than areas with phalangeal scars (non-flat epithelium). Scale bars are 25 μm. Sup = superior, Inf = inferior, Med = medial, Lat = lateral, P = pillar cell.

hESCs were transplanted into the scala media through a cochleostomy 7 days after the deafening surgery. Transplantation procedures did not affect the health of the animals, causing no morbidity or mortality. Guinea pigs were terminated at one of three different time points (6 hrs, 1 day or 7 days) after transplantation, and their cochleae were harvested for epi-fluorescence analysis of hESCs (identified by expression of GFP). Analysis at the 1 day time point revealed that hESCs were present along the epithelium of the cochlear duct in non-sensory regions such as Reissner’s membrane, lateral wall and spiral limbus (Fig. 3 top row) as well as in the sensory regions, as seen in areas with phalangeal scars (Fig. 3 middle row) or flat epithelium (Fig. 3 bottom row).

**Figure 4:**
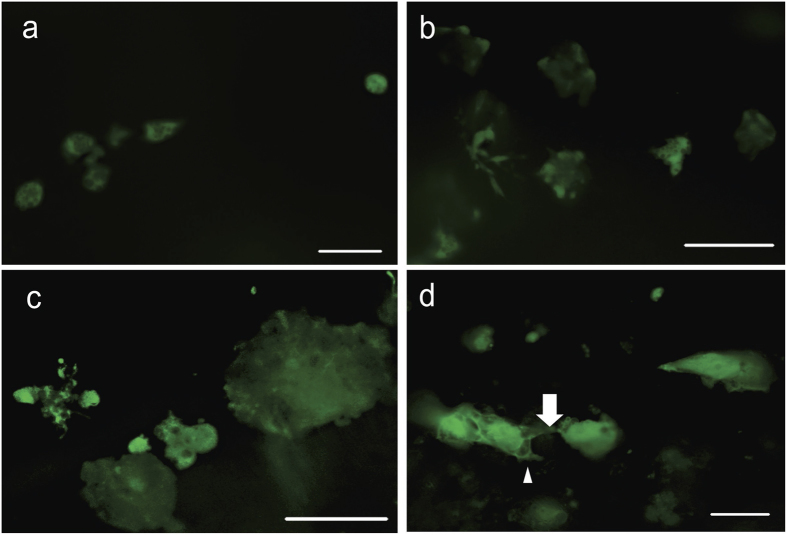
Shapes and distribution of transplanted hESCs. GFP-positive cells (green) observed 6 hours after injection were spherical (**a**) or irregular (**b**) in shape, and sometimes appeared to be merged into aggregates (**c**). At 1 day, some spherical cells showed cytoplasmic extensions (**d**) to other GFP-positive cells (arrow) or to adjacent GFP-negative areas (arrow head). Scale bars are 25 μm.

Various shapes of GFP-positive cells were observed in the cochlear duct. Cells were spherical (Fig. 4a) or irregular (Fig. 4b), and appeared as single cells or aggregates (Fig. 4c). At 1 and 7 days we observed spherical cells with cytoplasmic extensions (Fig. 4d) protruding toward other GFP-positive cells (arrow) or adjacent GFP-negative areas (arrowhead). These morphologies were quantitatively assessed in the entire sensory area (organ of Corti) and adjacent inner sulcus (these regions comprise 30% of the cross section of scala media).

The average number of GFP-positive cells in the region of the auditory epithelium was 203 cells/cochlea, which is about 1% of total transplanted hESCs. This number includes only cells in the sensory area and inner sulcus, the areas that could be whole-mounted for cell counting. Other areas of the cochlear duct included many other GFP-positive cells, but quantification was not performed due to the difficulty of obtaining flat tissue mounts of these areas. Qualitative observations suggest that GFP-positive cells were spread evenly throughout the cochlear duct (sensory and other areas); because the quantified area (sensory epithelium region) is about a third of the entire surface, we extrapolated the survival rate to 3.3% (average of three time points).

### Density of transplanted hESCs in the organ of Corti and inner sulcus

**Figure 5:**
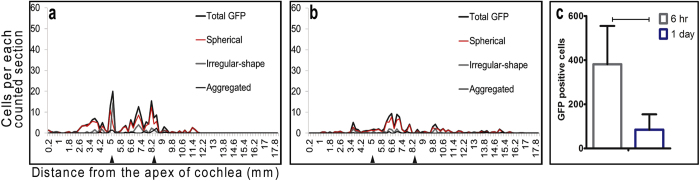
Density of transplanted hESCs in the organ of Corti and inner sulcus changed over time. All morphologies were widely distributed at both 6 hrs and 1 day (**b**), but the peak value on the cytocochleogram was reduced at 1 day (<10 cell/area). Total number of GFP-positive cells drastically declined between 6 hrs and 1 day (**c**) (p < 0.05).

The distribution of GFP-positive cells was evaluated by cytocochleogram (Fig. 5a,b) at two different time points (6 hrs and 1 day). At the 6-hr time point, all morphologies were widely distributed and the highest number of cells (>20 cell per 0.20 mm width) was observed at 5 mm away from apical end, which is near the cochleostomy used to introduce the cells (Fig. 5a). At the 1 day time point, a similar wide distribution pattern was observed, but the peak density was reduced (<10 cells per 0.20 mm width) (Fig. 5b). The total number of GFP-positive cells was significantly lower at 1 day (*p = *0.012) (Fig. 5c).

### The effect of sodium caprate on density and stemness of transplanted hESCs

**Figure 6:**
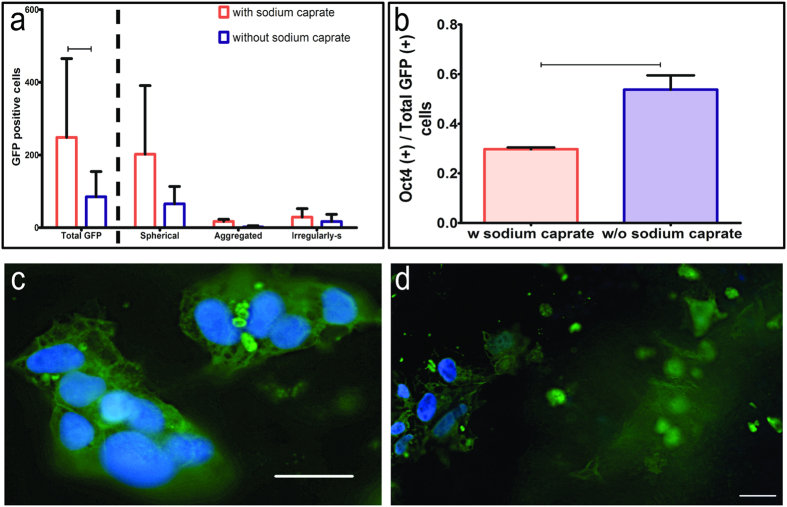
The effect sodium caprate on density and stemness of transplanted hESCs. (**a**) Number of GFP-positive cells was greater in cochleae flushed with sodium caprate (p < 0.05). (**b**) In the sodium caprate treated cochleae, the proportion of GFP-positive cells expressing the stem cell marker Oct3/4 was significantly lower. (**c**) A cochlea that was not flushed with sodium caprate, showing the high proportion of GFP-positive cells that were co-labelled with Oct3/4 in the nucleus. (**d**) A cochlea flushed with sodium caprate, showing a much smaller portion of GFP-positive cells co-labelled with Oct3/4. Scale bars are 25 μm.

Sodium caprate has been shown to transiently disrupt the junctional complexes of epithelial cells^13^,^26^. To enhance adhesion of the hESCs to the endogenous cells or facilitate insertion into the tissue, we added sodium caprate to the conditioning protocol. Transplantations were performed with or without sodium caprate, and animals were sacrificed at the 1-day time point. We then determined the density of the GFP-positive cells and assessed the proportion of Oct3/4-positive cells among GFP-positive cells for both treatment groups. The number of GFP-positive cells was greater in cochleae flushed with sodium caprate (*p* = 0.020) (Fig. 6a). The proportion of GFP-positive cells expressing the stem cell marker Oct3/4 was significantly lower (*p* = 0.048) (Fig. 6b). In cochleae that were not flushed with sodium caprate, there were fewer GFP-positive cells and a higher proportion of them were co-labelled with Oct3/4 in the nucleus (Fig. 6c). This result indicates that including sodium caprate in the conditioning procedure increases survival of the GFP-positive cells at the 1-day time point. Some of the cells had very large nuclei (nearly 25 μm), which may indicate swelling as part of a degenerative process or another response to the high potassium concentration in the extracellular fluid of their new environment.

### Distribution and density of transplanted hESCs (sodium caprate preconditioned) at 7 days

**Figure 7:**
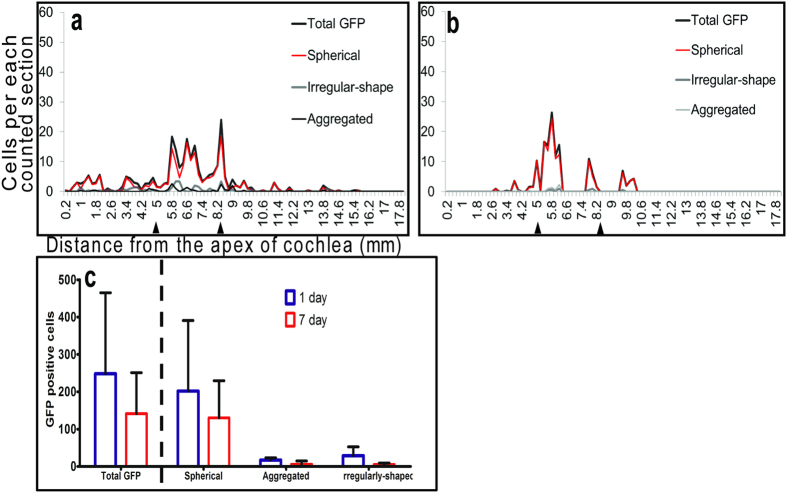
Distribution and density of transplanted hESCs at 7 days. All morphologies were widely distributed at 1 day (**a**), but had narrower distributions at 7 days (**b**). Total number of GFP-positive cells declined between 1 day and 7 days (**c**).

To test whether sodium caprate enhanced longer-term hESC survival, we extended the post-inoculation survival time to 7 days and assessed the distribution and density of the transplanted hESCs. All stem cell morphologies were widely distributed at 1 day (Fig. 7a), but had narrower distributions at 7 days (Fig. 7b). At the 7-day time point, GFP-positive cells were mostly observed near the cochleostomy site (5 mm away from apical end). The total number of GFP-positive cells was lower at 7 days than at 1 day (Fig. 7c), but the difference was not statistically significant (*p* = 0.489).

## Discussion

The goal of this study was to prepare cultures of hESCs and determine if they can survive long enough after injection into the conditioned scala media of deaf guinea pig cochleae to permit further biochemical manipulations leading to their differentiation into new hair cells. The cochleae were conditioned by administering a loop diuretic and flushing scala media with stem cell culture media and sodium caprate prior to the transplantation of the hESCs. We found that some of the injected hESCs survived for at least 7 days after the transplantation. Additionally, we found that sodium caprate, which was used for transient disruption of junctional complexes, facilitated the survival of hESCs and also increased the proportion of hESCs without Oct3/4, which may indicate post-implantation differentiation, as previously shown^27^.

Placing cells in a culture dish filled with artificial endolymph, which is potassium-rich, leads to their prompt demise^13^. At present, we can only speculate on the reasons for the rapid death of cells in endolymph-like fluids or in the endolymph of the scala media. Several factors likely contribute to cell death of exogenous, implanted cells exposed to endolymph. First, the high potassium environment in endolymph will shift the potassium equilibrium and depolarize the cell membranes. The result could favor uncontrolled calcium influx from voltage-gated calcium channels and impair volume regulation. Both effects could link chronic depolarization to perturbations in metabolic homeostasis, leading to cell death. Second, the process of dissociation alone causes cell death in ESCs and other cell types that are supported by cell-cell contact.

The ability of the cells to survive in the conditioned cochlea suggests that the cells can undergo gradual changes in homeostatic processes governing volume regulation and ion channel signaling. We speculate that one way to prevent cell death due to swelling or shrinking is to regulate passive rapid movement of water across the membrane, possibly by rearranging aquaporins and other channels in the membranes to accommodate the changing environment^28^. The conditioning measures we have implemented are transient, but recovery of potassium levels is slow as the stria vascularis regains functionality (with furosemide levels declining) and sodium is replaced with potassium in the scala media, giving the transplanted cells hours to implement the changes required by the new environment. It is possible that further optimization of the conditioning protocol, by changing timing and or altering other chemical parameters in the fluids may further increase hESC survival. However, the results reported here provide proof of the principle that a conditioning protocol can extend survival of relatively fragile cells such as hESCs in the scala media and provide a time window for further manipulations needed for inducing differentiation of the implanted cells into the desired phenotype. As such, the data we present demonstrate that transient conditioning is a useful first step towards practical transplantation of stem cells into the inner ear and that the time window between day 1 and 7 post-transplantation is suitable for initiating additional measures for inducing differentiation of these stem cells.

Pluripotency is an unstable cell state. The maintenance of pluripotency in cell culture is a well-studied though constantly evolving field. In general, pluripotency in stem cell cultures is maintained by feeder layers or the use of highly defined, feeder-free, serum-free culture media. In this study, hESCs were passaged in E8 medium, which contains known factors important for cell survival and maintenance of stemness, including insulin, FGF2, and TGF-beta^29^. For transplantation, the media was further supplemented with a Rho associated kinase (ROCK) inhibitor that supports survival of dissociated stem cells^30^,^31^. Our goal was to promote survival by flushing scala media with this formulation that includes salts similar to normal high-sodium extracellular saline solution. However, the exchange of endolymph for this pro-survival medium is certainly transient. While the rate of restoration toward a normal ionic environment is uncertain, the components of the exogenous medium are expected to degrade over time, metabolize, diffuse out of the cochlear duct, and/or absorb into cellular compartments. Thus, the loss of media components that inhibit differentiation and the subsequent exposure to other endogenous growth factors and contact cues within the cochlear duct should foster differentiation. Although the fate of differentiated cells in this study was not systematically evaluated, the survival of hundreds of cells offers the possibility that they could be subsequently guided to differentiate through successive developmental milestones by supplying exogenous factors, via timed drug release or cannulation. Cues can be taken from current protocols for *in vitro* reprogramming strategies and would necessarily include manipulation of BMP signaling to produce non-neural ectoderm and subsequent treatment with FGF and Wnt to influence otic differentiation^32^. Introduction of the appropriate exogenous factors will likely need to occur shortly after the surviving cells are stabilized in the scala media.

Integration of the transplanted cells into the auditory epithelium is important for their function after they differentiate into new hair cells, and likely also for their long-term survival. In the toxic environment of the endolymph, integration with the endogenous tissue may be especially important for survival of the transplanted cells. The inclusion of sodium caprate in the conditioning protocol was primarily intended to accomplish a transient degradation of the epithelial apical junctions and facilitate insertion and integration of the injected cells into the native epithelial layer^13^. Although partial integration has been demonstrated when HeLa cells were injected into conditioned scala media^13^ there was no evidence that the hESCs also integrated. The hESCs may have attached to the surface of the epithelium without full integration. However, the use of sodium caprate enhanced survival and promoted differentiation of hESCs after implantation even without clear evidence for their integration, suggesting that modulating cell junctions of the recipient epithelium can influence the outcome of the implantation.

Because integration into the epithelium was not obvious, it is unclear why sodium caprate flushing was beneficial. One possibility is that the release of junctional actin mediated establishment of cellular communication between hESCs and the native tissue, which resulted in enhanced survival and attachment. Cytoplasmic extensions from the GFP-positive cells, which are found at later time points, support the speculation that contact-mediated communication between the stem cells and the native epithelium occurred and may have contributed to the survival and/or differentiation of the stem cells. Alternatively, it is possible that sodium caprate acted on cell signaling, independent of cell contact and integration. Mechanistic studies indicate that sodium caprate can alter intracellular calcium signaling and phospholipase C activity^33^, suggesting that changes to major cell signaling pathways play important roles in survival of transplanted stem cells. Therefore, it also possible that sodium caprate promoted survival and/or differentiation independent of cell contact cues.

The morphology of cells that remained in the auditory epithelium a week after transplantation was unique and could indicate they were not perfectly healthy. However, cells with similar morphology were present at 1 day and 1 week, indicating this morphology is not necessarily indicative of a degenerative process. Future experiments will need to determine how to induce differentiation of these cells, starting with the time window between day 1 and 7 after the transplantation.

The use of sodium caprate to disrupt the tight barrier of the auditory epithelium raises the possibility of negative consequences from potassium leakage through the epithelium. Such an influx of potassium could be detrimental to survival of spiral ganglion neurons. However, our previous report found there was no significant reduction of spiral ganglion neurons after transient junction opening^13^ suggesting that including sodium caprate is safe for the surviving native cochlear tissue, in addition to enhancing positive outcomes for integration of transplanted cells into the deafened epithelium.

Stem cell transplantation runs the risk of tumorigenesis, and there is some evidence of teratoma formation in stem cell-transplanted cochleae^34^. However, the hESCs we injected into the conditioned scala media did not show signs of uncontrolled proliferation or teratoma formation after one week. Although the lack of proliferative response at this early time point is a positive outcome, it would be necessary to determine whether quiescence is maintained in longer-term survival of the implanted cells. Controlled differentiation of the implanted cells to the desired fates, which should be the next major goal to accomplish, would further alleviate the concern of tumor formation.

Clinical applicability of the procedures we employ here for stem cell implantation needs to be addressed. Delivering cells (or reagents) into the perilymph (scala tympani) is technically less challenging than injecting substances into the scala media. However, injecting cells into perilymph places the cells in the wrong side of the basilar membrane, where the potassium concentration is low thereby limiting the potential for sound transduction^35^,^36^. For this reason, injection into scala media appears to be a prerequisite, and the need to condition the cochlea to allow the slow adaptation of cells to the ionic environment in the endolymph can accomplish this task. It should be emphasized, however, that access to the scala media is difficult in laboratory animals and nearly impossible in the human ear with current technology.

Another hurdle for enhancing clinical use of this method is to accomplish differentiation of implanted cells into new hair cells, or other types of cells as needed based on the disease being treated. Several prior reports provide protocols for differentiating stem cells into hair cell-like cells in a dish^4^,^5^,^6^,^8^,^9^,^10^,^37^,^38^. The ability to manipulate cells once they are in the living cochlea is limited but not impossible. It is hoped that providing a few chemical guides via a mini-osmotic pump would combine with native signaling in the cochlea to guide differentiation of cells into the desired phenotype. The other task would be to “home” the implanted cells into a place where they will be physiologically useful. One way to accomplish this could be placement of ligands on their membranes, which will guide the cells to an area where the receptor to this ligand is expressed.

For new hair cells to function properly in the cochlea, it is necessary for the EP and potassium levels to recover (at least partly) following the conditioning procedure. We have shown that hearing can be recorded using ABRs in normal (non-deafened) animals that received furosemide injection and endolymph flush. This finding is in line with previous observations on the transient nature of stria vascularis pumps block by furosemide. For instance, reversible change of EP was seen in guinea pigs after furosemide and other diuretics^39^. Reversible changes in EP also have been demonstrated in the chinchilla, where a dose-related fall in EP was observed following injection of furosemide, and the levels of EP recovered after less than two hours^40^,^41^. Recovery of EP was noted even after furosemide was given at 100 times the typical human therapeutic dose^42^. In line with this experimental animal data, transient hearing loss was also found in humans after high dose furosemide treatment^43^. Together with our observation of partial recovery of hearing in non-deafened guinea pigs that received the conditioning protocol, these data suggest that stria vascularis functions recover after this treatment, suggesting that the conditioning itself is not detrimental for continued function of the pumps.

Hair cell generation is the ultimate goal of the stem cell therapy for the auditory epithelium, but connections to the neurons are essential if these cells were to contribute to hearing. Once stem cells can be guided to survive, integrate in the right place, and differentiate to the hair cell phenotype, it will be necessary to connect them with the neural substrate of the inner ear. If cells are engineered to secrete neurotrophins they may be able to attract nerve endings, because auditory neurons can grow towards cells that secrete neurotrophins^44^. Formation of synaptic connections would be the next step for functional activation of action potentials in the auditory nerve. It is not easy to envision an ability to recreate the complex organ of Corti by implanting stem cells into a deaf ear. However, it is hoped that placing a population of stem cells that can become new inner hair cell-like cells, connect to the neurons and initiate their firing would bestow some functional hearing in profoundly deaf ears, allowing the plasticity of the brain to accommodate and decipher the new signals.

In conclusion, we determined that a set of “conditioning” procedures aimed at transiently reducing the concentration of potassium in the scala media facilitate survival of hESCs in the endolymph. Cells survived in the scala media for at least one week after transplantation. Some of the stem cells lost Oct3/4 expression suggesting loss of pluripotency and possibly initiation of differentiation. Although major obstacles for generating new hair cells from stem cells injected into the living ear remain, survival of cells in host tissue clears the way to the next task, which is the design of ways to complete the differentiation of the stem cells once they are in the inner ear.

## Materials and Methods

### Cells and culture

A modified hESC line (H9 Cre-LoxP, Lot WA09(LOXGFP)-MCB-01, WiCell Research Institute, passages 22 to 42) was used for transplantation. In these cells, a floxed humanized recombinant GFP (hrGFP) transgene is constitutively expressed, sustained during differentiation, and suitable for tracing human cells in xenografts. hESC were maintained on hESC certified Matrigel^®^ (Corning 354277) coated standard tissue culture plates (Corning) in TeSR-E8 media (Stem Cell Technologies 05940) and passaged by dissociation with dispase (2 mg/ml in DMEM/F12). Stemness was monitored by morphology of hESC colonies and immunostaining for the pluripotency marker Oct3/4. For transplantation, hESC colonies were dissociated to single cells with trypsin-EDTA (0.25%, life technologies) aided by manual dissociation in PBS containing defined trypsin inhibitor (Gibco) and filtering through a 40 μm cell strainer (Corning). After centrifugation (5 min, 300 g), the cell pellet was suspended in TeSR-E8 supplemented with Y-27632 Rho kinase inhibitor (10 μM, Calbiochem). Cells were counted and concentrated to 20,000 cells/μl by centrifugation (1.5 min 600 g) and resuspension in TeSR-E8 containing Y-27632. The single cell suspension was chilled on ice and 2 μl used for transplantation.

For immunostaining, colonies were passaged onto Matrigel-coated 15 mm plastic coverslips (Thermo Fisher Scientific) and fixed after 3 days. Single cells were plated onto 13 mm plastic cover slips and fixed after 1 h, 1 day, or 3 days of culture in either TeSR-E8 or serum free DPM (DMEM/F12 containing HEPES supplemented with GlutaMAX (1x), non-essential amino acids (1x), N2 (1x) and B27 (1x)). At the time of collection, cells were briefly washed with DPBS and fixed with 4% PFA in DPBS (10 min), blocked and permeabilized in 5% normal donkey serum in DPBS containing 0.1% Triton-X 100 and stained with anti-Oct3/4 (1:100, Santa Cruz Biotechnology SC-8628) and anti-hrGFP (1:200, Agilent 240141) overnight at 4 °C. Coverslips were washed, stained with Alexa Fluor 568 and 488 secondary antibody (1:500, Invitrogen) for 1 h at room temperature, counterstained with Hoechst (1:10 000, 5 min) to label nuclei, and mounted with Prolong Gold. For evaluation of Oct3/4 expression 9 random regions of interest per coverslip were imaged on a BX51 W microscope (Olympus) outfitted with an ORCA-Flash4.0 CMOS camera (Hamamatsu), and counts of Oct3/4-positive cells as a percentage of total cells per field were averaged across a minimum of 3 independent preparations per condition.

### Animals

**Figure 8:**
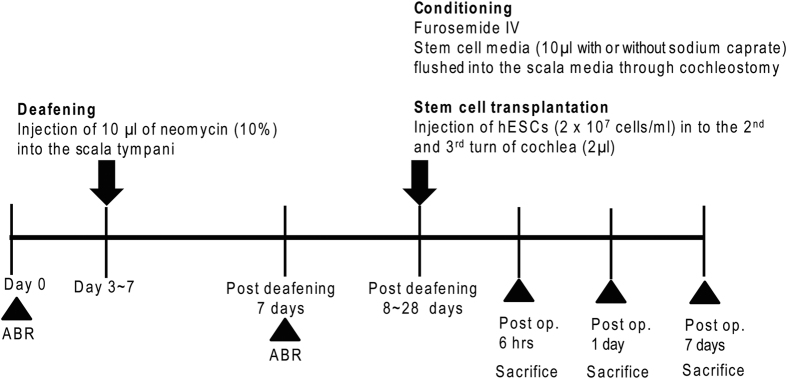
Schematic time line of *in vivo* cell transplantation experiment. At least 8 days after the initial deafening surgery, the cochleae were preconditioned by i.v. furosemide injection and flushing of scala media with stem cell culture media. Additionally, endolymph was flushed with or without sodium caprate dissolved in stem cell culture media. After the preconditioning procedure, hESCs (GFP fluorescence labelled) were implanted in the scala media through the cochleostomy.

Eighteen pigmented, specific pathogen free (SPF) guinea pigs from the KHRI SPF Colony were used for this study. The animals were between one and two months old (250–400 g) at the beginning of the experiment. This study was approved and performed according to the guidelines of the University of Michigan Institutional Animal Care and Use Committee (IACUC). Prior to performing experimental procedures, hearing function was evaluated by ABR to establish baseline thresholds. All animals were deafened with neomycin (see below) 3 to 7 days after the ABR test. After the deafening, ABR thresholds were re-evaluated at least 1 week after the initial surgery. Stem cell transplantation surgery was performed between 8 and 28 days after deafening surgery with prior conditioning of cochlea (Fig. 8). The animals were sacrificed at different time points after the transplantation surgery; 6 hrs (n = 3), 1 day (n = 10) and 7 days (n = 3). Two additional animals received neomycin deafening but no transplantation related procedure and were sacrificed 7 days later to evaluate the histologic outcome of neomycin deafening. Two other animals were not deafened but received the conditioning protocol (furosemide IV and flushing endolymph with artificial perilymph), allowed to survive for 4 days to assess effects of the conditioning protocol on hearing and cochlear histology. Measurement of hearing thresholds by ABR in these guinea pigs was used to indirectly confirm at least partial recovery of EP after the conditioning procedure.

### ABR measurement

ABR recordings were collected at 8, 16 and 32 kHz in the left ear of each animal. Animals were anesthetized intramuscularly with ketamine (58.8 mg/kg), xylazine (2.4 mg/kg) and acepromazine (1.2 mg/kg) and placed on a thermo-regulating heating pad to maintain body temperature. ABRs were recorded in an electrically and acoustically shielded chamber (Acoustic Systems, Austin, TX USA). Tucker Davis Technologies (TDT) System III hardware and SigGen/BioSig software (TDT, Alachua, FL USA) were used to present the stimulus and record responses. Neural activity in response to brief tone bursts were measured using needle electrodes inserted subcutaneously ventral to each pinna and at the vertex of the skull. The sound was delivered to an area just inside the tragus. Each tone burst was 15 ms in duration, with 1 ms rise/fall times, presented 10 bursts per second through an EC1 driver (TDT, aluminum enclosure made in-house). Up to 1024 responses were averaged for each stimulus level and each frequency. Responses were collected for stimulus levels in 10 dB steps at higher stimulus levels, and at 5 dB steps near threshold. Thresholds were interpolated between the lowest stimulus level where a response was observed, and 5 dB lower, where no response was observed. For statistical analysis of ABR data, multivariate analysis of variance (MANOVA; IBM SPSS software, version 21.0) was used to test for changes in threshold after deafening surgery. Subsequently, univariate ANOVA was performed to evaluate threshold differences between time points for each frequency, with sequential Bonferroni criterion used to correct for the number of post-hoc tests.

### Surgeries (unilateral deafening and stem cell transplantation)

As for the deafening procedure, animal was anesthetized using ketamine HCl 40 mg/kg (Ketaset, Fort Dodge Animal Health, Fort Dodge, IA) and xylazine 10 mg/kg (AnaSed, Shenandoah, IA) and surgical area was prepared for the sterile surgery. The cannula used for injecting the regents to the inner ear was prepared prior to surgery, as follows: a polyethylene tube connected to a fine polyimide tube at one end, and to a 10 μl Hamilton syringe with a needle filled with sterile water at the other end. The animals were placed in the lateral position. 0.5 ml of 1% lidocaine HCl was injected subcutaneously in the post-auricular area for local anesthesia. Retroauricular incision was made and muscle beneath skin and above bulla was retracted. After visualization of the bulla, a small opening was made to approach the basal turn of cochlea. The cochleostomy was made near the round window using a hand drill. 10 μl of 10% neomycin solution was diffused into the scala tympani using Hamilton syringe and micropump. A piece of muscle was placed over the cochlea opening and the bulla was closed using bone cement. After layer-by-layer suture, animal was recovered.

Between 8 and 28 days after the initial surgery, animals were anesthetized and prepared with same protocol. The animals were placed in the supine position. 0.5 ml of 1% lidocaine HCl was injected subcutaneously in the submandibular area for local anesthesia. A submandibular incision was made and furosemide (60 mg/kg, Hospira Inc., Lake Forest, IL) was injected into the jugular vein. The muscle between mandibular bone and jugular vein was retracted to expose the ventral surface of the bulla and a small opening was made in the bulla to approach the cochlea. Using the dark stria vascularis as a landmark, scala media was opened by cochleostomies in the second turn and third turn. Experimental substances where introduced through the second turn cochleostomy and excessive fluid was allowed to escape from the third turn. First, 2 μl of stem cell media (TeSR-E8 supplemented with 10 μM Y-27632, see stem cell procedures) or media with 10 mmol/l sodium caprate (Sodium decanoate, Sigma-Aldrich Co., St. Lois. MO) were injected into scala media. As noted above, some animals received only these fluids to assess their effects on native tissues, in others, these fluids were followed by 2 μl of human stem cells (~20 million/ml). Volume and rate of injection were using a Hamilton syringe and stereotactic micropump. After the injection, pieces of muscle were placed over the two cochleostomies. The bulla opening was closed using bone cement and incision was sutured layer by layer. The procedures took approximately 45 minutes to complete. Afterwards, the animals were allowed to awaken from anesthesia, hydrated with subcutaneous injection of normal saline and their pain was controlled with carprofen (4 mg/kg, s.c., Rimadyl, New York, NY).

### Tissue-preparation and Immunocytochemistry

Animals were sacrificed at three different time points (6 hrs, 1 day and 7 days) and their temporal bones were harvested. Samples were fixed in 4% paraformaldehyde in phosphate buffered saline (PBS) for at least 2 hours, rinsed with PBS, and dissected for whole-mount preparations. The dissected tissues were permeabilized in 0.3% Triton X-100 in PBS for 10 min, then incubated with blocking buffer (5% normal goat serum in PBS) for 30 min to block non-specific binding of secondary antibodies. After that, samples were incubated with primary antibody; Oct3/4 Antibody (Santa Cruz Biotechnology, Inc., Dallas, TX, see stem cell procedures). After rinsing the primary antibody with PBS, tissues were incubated with a fluorescence-labeled secondary antibody for 30 min and rinsed with PBS before mounting on microscope slides. The secondary antibodies used for Oct3/4 Antibody was Alexa Fluor^®^ 350 (Invitrogen/Molecular Probes, Carlsbad, CA) diluted 1:200 in PBS, incubated for 30 minutes. To label F-actin, permeabilized dissected tissues were incubated with Alexa Fluor 488-conjugated phalloidin (1:400 dilution in PBS, 30 min, room temperature). After PBS rinsing, stained tissues were mounted on glass slides with Fluoro-Gel mounting media (Electron Microscopy Sciences, Hatfield, PA). For epi-fluorescence analysis, we used a Leica DMRB epi-fluorescence microscope (Leica, Eaton, PA) equipped with a SPOT-RT digital camera (Diagnostic Instruments, Sterling Heights, MI) and SPOT-RT software Ver.5.0.

### Assessment of GFP-positive cells

GFP-positive cells were evaluated in whole-mounted tissue, which contains 30% of total scala media area from a cross section image of the cochlea. We counted all the GFP positive configurations in the cochlea form base to apex using the fluorescence microscope. To generate cytocochleograms of GFP-positive cells, tissues were viewed in a Leica DMRB epi-fluorescence microscope with 40x (1.25x digital zoom) objective lens. GFP-positive cell counts were analyzed modifying the KHRI Cytocochleogram Program, Version 3.0. In each field of view, a 0.20 mm scale which was placed in the 10x eyepiece was adjusted along the centers of the pillar cells or flat epithelium. The number of GFP-positive cells for each 0.20 mm segment was calculated for each row. To compare between specimens differing in total cochlear length, each specimen’s data were used to calculate the percent of hair cell loss in 75 intervals (each representing 1.33% of that specimen’s cochlea). Total number of GFP-positive cells in entire whole mounted specimen (apex to base) was assessed and compared; number of cells with each different shape was counted as well. Group differences in total number of GFP-positive cells were evaluated by student T-test and comparisons in IBM SPSS software, version 21.0; *p *<* 0.05* was considered significant.

## Additional Information

**How to cite this article**: Lee, M. Y. *et al*. Survival of human embryonic stem cells implanted in the guinea pig auditory epithelium. *Sci. Rep.* **7**, 46058; doi: 10.1038/srep46058 (2017).

**Publisher's note:** Springer Nature remains neutral with regard to jurisdictional claims in published maps and institutional affiliations.

## Supplementary Material

Supplementary Figure 1
