# Supplementary material for: Survival of human embryonic stem cells implanted in the guinea pig auditory epithelium
Source: Sci Rep. 2017 Apr 7;7:46058. doi: 10.1038/srep46058 (PMC5384248; doi:10.1038/srep46058)

Supplementary information for:

Survival of human embryonic stem cells implanted in the guinea pig auditory epithelium

Min Young Lee, Sandra Hackelberg, Kari L. Green, Kelly G. Lunghamer, Takaomi Kurioka, Benjamin Loomis, Donald L. Swiderski, R. Keith Duncan, Yehoash Raphael

Supplementary Figure 1. whole-mount of the auditory epithelium from a non-deafened ear that received the conditioning treatment

Epi-fluorescence view of phalloidin stained organ of Corti from a non-deafened guinea pig that received the conditioning protocol. A. an area apical to the region of the injection site, the morphology is normal and only one hair cell is missing. B. In an area closer to the site of injection, a massive loss of outer hair cells is seen, but most inner hair cells survive. Scale bar is 40 μm.

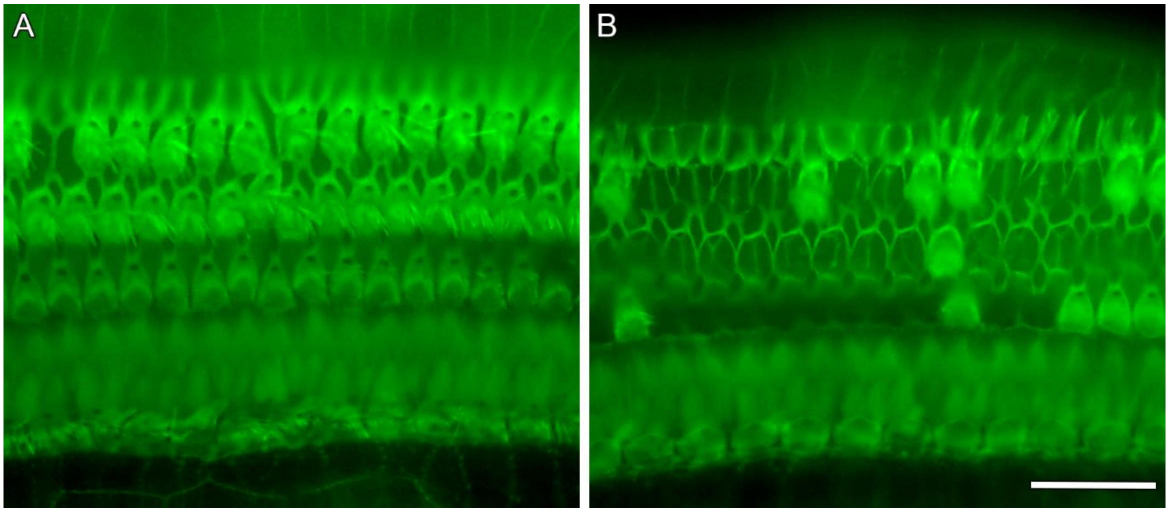

Supplement: Supplementary Figure 1 [file srep46058-s1.pdf]
